# Intranodal administration of mRNA encoding nucleoprotein provides cross-strain immunity against influenza in mice

**DOI:** 10.1186/s12967-019-1991-3

**Published:** 2019-07-25

**Authors:** Patrick Tjok Joe, Ioanna Christopoulou, Lien van Hoecke, Bert Schepens, Tine Ysenbaert, Carlo Heirman, Kris Thielemans, Xavier Saelens, Joeri L. Aerts

**Affiliations:** 1 Laboratory for Molecular and Cellular Therapy, Department of Biomedical Sciences, Vrije Universiteit Brussel (VUB), Jette, Belgium; 2 VIB-UGent Center for Medical Biotechnology, VIB, Ghent, Belgium; 3 Department for Biomedical Molecular Biology, Ghent University, Ghent, Belgium; 4 Department of Biochemistry and Microbiology, Ghent University, Ghent, Belgium; 5 Laboratory of Pharmaceutical Biotechnology and Molecular Biology, Vrije Universiteit Brussel, Laarbeeklaan 103D, 1090 Brussels, Belgium

**Keywords:** mRNA, Influenza, Universal vaccine, Intranodal, Nucleoprotein, T cell

## Abstract

**Background:**

Current human influenza vaccines lack the adaptability to match the mutational rate of the virus and therefore require annual revisions. Because of extensive manufacturing times and the possibility that antigenic alterations occur during viral vaccine strain production, an inherent risk exists for antigenic mismatch between the new influenza vaccine and circulating viruses. Targeting more conserved antigens such as nucleoprotein (NP) could provide a more sustainable vaccination strategy by inducing long term and heterosubtypic protection against influenza. We previously demonstrated that intranodal mRNA injection can induce potent antigen-specific T-cell responses. In this study, we investigated whether intranodal administration of mRNA encoding NP can induce T-cell responses capable of protecting against a heterologous influenza virus challenge.

**Methods:**

BALB/c mice were immunized in the inguinal lymph nodes with different vaccination regimens of mRNA encoding NP. Immune responses were compared with NP DNA vaccination via IFN-γ ELISPOT and in vivo cytotoxicity. For survival experiments, mice were prime-boost vaccinated with 17 µg NP mRNA and infected with 1LD50 of H1N1 influenza virus 8 weeks after boost. Weight was monitored and viral titers, cytokines and immune cell populations in the bronchoalveolar lavage, and IFN-γ responses in the spleen were analyzed.

**Results:**

Our results demonstrate that NP mRNA induces superior systemic T-cell responses against NP compared to classical DNA vaccination. These responses were sustained for several weeks even at low vaccine doses. Upon challenge infection, vaccination with NP mRNA resulted in reduced lung viral titers and improved recovery from infection. Finally, we show that vaccination with NP mRNA affects the immune response in infected lungs by lowering immune cell infiltration while increasing the fraction of T cells, monocytes and MHC II^+^ alveolar macrophages within immune infiltrates. This change was associated with altered levels of both pro- and anti-inflammatory cytokines.

**Conclusions:**

These findings suggest that intranodal vaccination with NP mRNA induces cross-strain immunity against influenza, but also highlight a paradox of influenza immunity, whereby robust immune responses can provide protection, but can also transiently exacerbate symptoms during infection.

**Electronic supplementary material:**

The online version of this article (10.1186/s12967-019-1991-3) contains supplementary material, which is available to authorized users.

## Background

The development of an influenza vaccine in the late 1940s can be considered as one of the great milestones in modern medicine. Currently licensed human influenza vaccines are very safe and likely prevent numerous deaths and hospitalizations [1]. In general, protection provided by conventional influenza vaccines depends upon the generation of neutralizing antibody responses against the viral hemagglutinin (HA) and, to some extent, antibodies that can inhibit the viral neuraminidase (NA) activity. Such responses can effectively reduce or even prevent influenza and confer herd immunity on a population scale but are largely restricted to the strains included in the vaccine. Mismatches between the strains covered by the vaccine and the strains that are actually circulating can therefore result in a reduced, if not abolished, efficacy of the vaccine leading to an increased disease burden, and therewith associated a higher mortality, but also a significant financial cost for companies and society [2, 3].

Influenza vaccine formulations require annual revisions due to the relatively high mutational frequencies that occur within the major antigenic regions of HA and NA through the processes of antigenic drift [4]. This requirement, together with a significant production time of several months, forces vaccine manufacturers to rely on predictions of the strain that will most likely circulate in the next season, thereby running the risk of developing a mismatched vaccine [5].

For this reason, and, for influenza A, as a measure against a future pandemic influenza outbreak, the development of a so-called universal vaccine that targets more conserved antigens, such as the nucleoprotein (NP), could provide a more sustainable approach by protecting against multiple strains of influenza [6]. The presence of T cells that target conserved antigens, and are capable of lysing virus-infected cells, has been demonstrated to correlate with reduced disease and enhanced viral clearance in influenza patients [7, 8]. In addition, pre-existing CD8^+^ T-cell responses against NP have been shown to cross-react with multiple subtypes of influenza, highlighting the breadth of protection that could be achieved through T-cell based vaccines [9–12].

Several studies have established proof of concept for the development of a universal vaccine using gene-based strategies such as the use of DNA plasmids or viral vectors. However, such delivery methods still have important drawbacks regarding safety and clinical efficacy. In contrast, mRNA-based gene delivery strategies offer a more favorable safety profile as exogenous mRNA is only transiently present in the cytosol and thus unlikely to incorporate into host genes [13]. In addition, mRNA possesses an intrinsic adjuvant activity that can activate toll-like receptors (TLRs) and RIG-I-like receptors (RLRs) [14]. Therefore, mRNA could provide an attractive platform for the development of a universal vaccine against influenza.

Previously, we have shown that intranodally delivered naked mRNA is almost exclusively taken up and translated by dendritic cells (DCs) [15]. DCs play a crucial role in T-cell activation through antigen-presentation and by providing immunostimulatory signals. Uptake of tumor antigen-encoding mRNA by these DCs resulted in robust T-cell responses that delayed tumor growth in mice.

Furthermore, intranodal vaccination was shown to allow for lower vaccine doses while maintaining the same immunogenicity as other administration routes [16–18]. Therefore, intranodal vaccination provides an attractive method for mRNA vaccines.

In this study, we evaluated whether intranodally delivered naked mRNA can elicit robust T-cell responses against the conserved antigen NP of an H3N2 influenza strain and compared these responses to those obtained through DNA vaccination.

## Materials and methods

### Animals

Specific pathogen-free female 6 week old BALB/c mice were purchased from Charles River, housed in individually ventilated cages and handled according to the guidelines and regulations of the animal care committee of the Vrije Universiteit Brussel (VUB, License number LA1230214, ethicom nr. 16-214-10 and institutional Ethics Committee on experimental animals of the Vlaams instituut voor Biotechnology (VIB, License Number LA1400536, ethicom nr.EC2014-076).

### In vitro transcription of mRNA vaccines

The sequence for NP was derived from the influenza A/NL/18/94 H3N2 nucleoprotein as described previously [19]. The NP sequence was cloned in-frame between the murine signal sequence of the murine LAMP-1 protein and the human transmembrane and cytoplasmic domains of human DC-LAMP, a subcellular targeting strategy to enhance presentation in both MHC I and MHC II class pathways [20, 21]. The sequence of hemagglutinin (HA) was derived from influenza A/PR/8/34 H1N1. Both HA and NP sequences were further cloned into the pEtheRNA vector (eTheRNA immunotherapies) containing a 5′ end translation enhancer and 3′ end RNA stabilization sequence. Truncated nerve growth factor (tNGFR) mRNA was produced as previously described [22].

The mRNA was then produced as described [15] and resuspended in 10 µl total volume per injection containing 2 µl endotoxin free water and 8 µl Hartmann (Baxter) solution following immunization.

### DNA plasmid preparation

The NP sequence from A/NL/18/94 H3N2 was cloned into the pCAXL plasmid vector and sequence verified [23]. LPS-free plasmid DNA vaccines were prepared following the manufacturer instructions (EndoFree Plasmid Kit, Qiagen). Endotoxin levels were subsequently determined using the Genscript ToxinSensorTM Chromogenic LAL Endotoxin Assay Kit.

### Immunizations

Intranodal immunizations with mRNA were performed by surgically exposing the inguinal lymph node of anaesthetized animals followed by the injection of the indicated amounts of mRNA in a total volume of 10 µl. Incision wounds were subsequently closed and disinfected. Repeated immunizations were alternated between the left and right inguinal lymph nodes. Mice in the control group received mRNA encoding tNGFR as previously described [15].

DNA vaccines were diluted in buffered saline to a concentration of 1 mg/ml and injected in the quadriceps muscles of the hind legs (50 µl/leg) of anesthetized mice.

### Viral challenges

Mice were sedated with isoflurane and infected intranasally with 1LD_50_ of influenza A Puerto Rico/8/1934 H1N1 (PR8). Body weight was monitored each day for 2 weeks. At 75% of the initial body weight, mice were sacrificed by cervical dislocation.

### Bronchoalveolar lavage

Bronchoalveolar lavage fluids (BALF) were isolated as described [24]. Mice were anesthetized and a small incision was made in the trachea to insert a lavage cannula in the trachea. Lungs were lavaged four times with 1 ml of HBSS with 0.05 mmol/l EDTA (Sigma-Aldrich). The first lavage fluid was used to determine cytokine levels. The BAL fluid was separated from the BAL cell pellet by centrifugation (7 min; 400*g*; 4 °C).

### Plaque assay

Tenfold serial dilution sera of BALF were added to a monolayer of MDCK cells in 6-well plates. MDCK were cultured in serum free OptiMEM medium (Invitrogen) supplemented with penicillin and streptomycin. After 1 h at 37 °C, serum free medium containing 0.6% avicel RC-851 (FMC Biopolymers) and trypsin was added to the cells for 3 days at 37 °C. After infection, cells were washed with PBS and subsequently fixed in 4% paraformaldehyde for 30 min at 37 °C. Plates were then washed with PBS and blocked for 1 h with 1% BSA and 0.05% Tween20 in PBS. To stain the viral plaques, convalescent anti-PR8 mouse serum was added for 1 h. After washing three times with 1% BSA in PBS, wells were incubated with HRP-conjugated anti-mouse IgG antibodies (Southern Biotech) for 1 h. Non-binding antibodies were removed by two washing steps with PBS containing 1% BSA and 0.05% Tween20 and one wash with PBS. Finally, TrueBlue peroxidase substrate (KPL, Gaithersburg) was added to visualize the plaques. The plaques of at least two different dilutions were counted and for each dilution, the number of PFU were calculated by multi-plying the number of plaques present at the given dilution with the corresponding dilution factor and expressed as the number of PFU/1 ml BAL fluid.

### IFN-γ ELISPOT

Isolated splenocytes were stimulated at 4 × 10^5^ cells per well (Multiscreen-IP PVDF Filter plates, Milipore) for 20 h with 5 µg/ml of peptides (Eurogentec). The NP peptide (147–155) is the cross-reactive H-2K^d^-restricted epitope derived from the influenza A H1N1 PR8 virus comprising the amino acid sequence TYQRTRALV and is sequence identical between influenza A H1N1 PR8 and influenza A/NL/18/94 H3N2. This peptide was shown to constitute the immunodominant epitope of NP in BALB/c mice [25].

The HA peptide (518–526) is a H-2K^d^-restricted epitope derived from the influenza A H1N1 PR8 virus comprising the amino acid sequence IYSTVASSL. For intra-assay negative and positive controls, medium (Mock) or Dynabeads Mouse T-Activator CD3/CD28 beads (Thermofisher, data not shown) were used, respectively. IFN-γ detection was performed using the murine IFN-γ ELISPOT kit from Diaclone. Spot forming cells (SFC) were counted using an ELISPOT reader (Autoimmun Diagnostika GmbH, Germany) and are shown after background (mock conditions) subtraction.

### In vivo cytotoxicity assay

The in vivo cytotoxicity was performed as previously described [26]. Splenocytes isolated from naïve BALB/c mice were used as target cells and pulsed for 90 min at 37 °C with 5 µg/ml of peptides before labelling the cells with 1.5 µM of CellTrace Violet (CTV, Thermofischer Scientific) according to the manufacturer’s protocol. Labeled, peptide-pulsed target cells were then mixed in a 1:1 ratio with non-pulsed splenocytes from naïve BALB/c mice labelled with 0.15 µM CTV according to the manufacturer’s protocol. The splenocyte mix was then resuspended in PBS and 1.5–2 × 10^7^ splenocytes were injected intravenously per animal. Lysis of target cells was analyzed 18 h later by flowcytometry of splenocytes isolated from the receiver mice (LSR Fortessa, Beckton Dickinson). Vehicle injected mice were used as a background control (non-immunized). Specific lysis was calculated as follows:

$$100 \, \times \, \left[ { 1- \left( {{{\left( {{{\% {\text{CTV}}^{\text{high}} } / {\% {\text{ CTV}}^{\text{low}} }} \, } \right)_{\text{immunized}} } \mathord{\left/ {\vphantom {{\left( {{{\% {\text{CTV}}^{\text{high}} } \mathord{\left/ {\vphantom {{\% {\text{CTV}}^{\text{high}} } {\% {\text{ CTV}}^{\text{low}} }}} \right. \kern-0pt} {\% {\text{ CTV}}^{\text{low}} }} \, } \right)_{\text{immunized}} } {\left( {{{\% {\text{ CTV}}^{\text{high}} } / {\% {\text{ CTV}}^{\text{low}} }}} \right)_{{{\text{non}} - {\text{immunized}}}} }}} \right. \kern-0pt} {\left( {{{\% {\text{ CTV}}^{\text{high}} }/ {\% {\text{ CTV}}^{\text{low}} }}} \right)_{{{\text{non}}{-}{\text{immunized}}}} }}} \right)} \right].$$

### Differential BAL cell counts

Flow cytometry was used to determine the number and types of cells present in the BAL fluid. Fc-blocked (1 µg/ml; eBiosciences) BAL cells were stained with anti-mouse SiglecF-phycoerythrin (PE; 1 µg/ml; BD Pharmingen), CD45- allophycocyanin (APC; 1 µg/ml; eBiosciences), CD3-PECy5 (1 µg/ml; eBiosciences), CD19-PECy5 (1 µg/ml; eBiosciences), CD11c-PECy7 (1 µg/ml; eBiosciences), MHCII-APC-efluor780 (1 µg/ml; eBiosciences), CD11b-V450 (1 µg/ml; BD Pharmingen), Ly6C-FITC 1 µg/ml; BD Pharmingen) and Ly6G-AlexaFluor700 (1 µg/ml; BD Pharmingen). Fixable Viability Dye eFluor^®^ 506 (eBiosciences) was added to exclude dead cells from the analysis.

### Luminex

Undiluted isolated BAL samples were mixed with a 10% BSA solution to obtain a final concentration of 0.5% BSA per sample. The assay was further performed according to the manufacturer’s instruction (Bio-Plex PRO™ Mouse cytokine 23-plex Assay, Bio-Rad) and samples were analyzed in duplicates on a Bio-Plex 200 system (Bio-Rad).

### Statistical analysis

Statistical analysis was performed using Graphpad Prism software version 6.00. Data was first analyzed for normal distribution using the Kolmogorov–Smirnov test. For normally distributed data, the unpaired two-tailed Student’s *t* test and one-way ANOVA test with Bonferroni correction were used for pairwise or multiple comparisons. In case of non-normally distributed data or small sample sizes, the non-parametric Mann–Whitney test was used for pairwise comparisons. For all analyses, the minimal level of significance was set at P < 0.05 (*).

## Results

### Intranodal immunization with mRNA induces stronger T-cell responses compared to intramuscular DNA vaccination

**Fig. 1:**
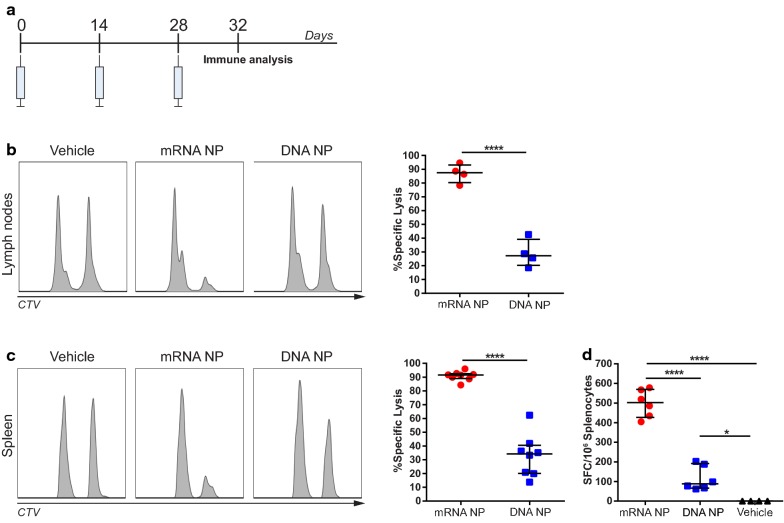
Comparison of T-cell responses after intranodal immunization with NP mRNA or intramuscular immunization with NP DNA. **a** BALB/c mice were immunized every 2 weeks for a total of three immunizations with 50 µg of intranodally delivered mRNA encoding NP, 100 µg intramuscularly delivered DNA encoding NP or 0.8 RL intranodally (vehicle). Analysis of in vivo cytotoxic T-cell responses (**b**, **c**) and ex vivo IFN-γ production (**d**) was assessed 4 days after the last immunization. **b**, **c** In vivo antigen-specific lysis of CellTrace Violet (CTV)-labelled NP peptide-loaded target cells was assessed in lymph nodes (**b**) and spleens (**c**). Histograms show the lysis of target cells and are representative for each group. **d** Ex vivo IFN-γ responses assessed by ELISPOT from splenocytes isolated from vaccinated animals and re-stimulated with NP peptide. Results from each experiment are shown as median ± interquartile range (IQR) with each symbol representing one mouse for a total of 4–8 mice per experiment. Differences between groups were analyzed by Mann–Whitney U test (**b**), Student’s *t* test (**c**), or one-way ANOVA (**d**)

Vaccination with DNA encoding conserved influenza antigens has been studied extensively and was shown to induce cross-protective immunity in small animals [27]. To assess the capability of intranodally administered mRNA to induce cytotoxic T-cell responses, we compared this vaccination method with the frequently used method of intramuscular DNA immunization. To this end, 50 µg of mRNA encoding NP derived from influenza A H3N2 or 100 µg of DNA encoding the same antigen were injected intranodally or intramuscularly, respectively. Immunizations were given every 2 weeks for a total of 3 immunizations (Fig. 1a).

In mice that received intranodal NP mRNA injections, a significant increase in the ability to lyse antigen-loaded target cells in lymph nodes and spleens compared to animals immunized intramuscularly with DNA was observed (Fig. 1b, c). Consistent with this finding, splenocytes from mRNA vaccinated mice showed significantly higher numbers of IFN-γ producing T cells specific for NP compared to DNA NP animals (Fig. 1d).

These data demonstrate that intranodal immunization with NP mRNA induces systemic T-cell responses against NP which are more potent than those induced by intramuscular DNA vaccination.

### Intranodal immunization with a low-dose mRNA vaccine induces prolonged T-cell responses against NP

**Fig. 2:**
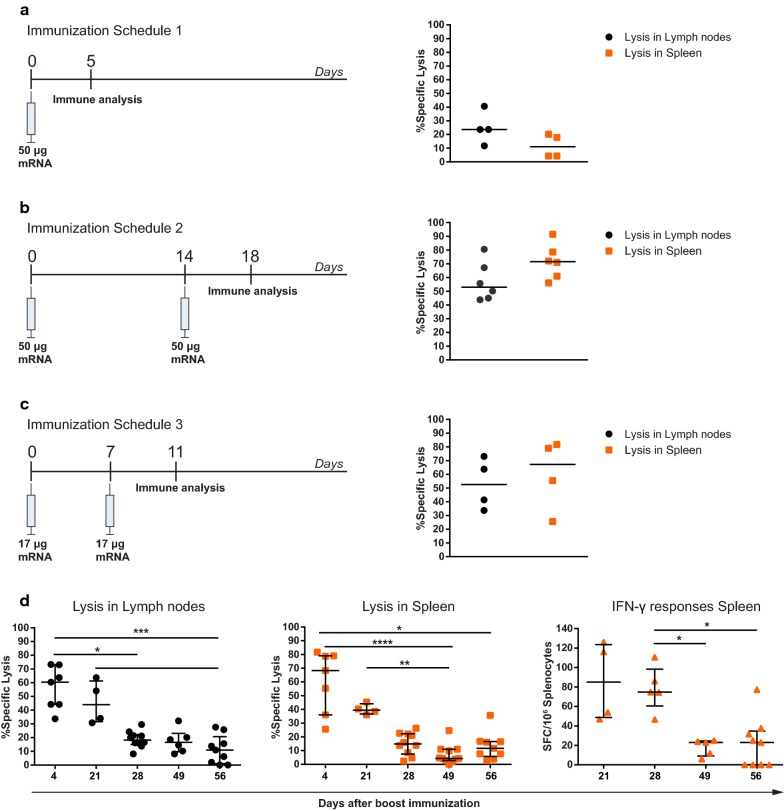
Evaluation of different immunization schedules and duration of T-cell responses after vaccination with NP mRNA. **a**–**c** BALB/c mice were immunized according to different vaccination schedules with varying amounts of mRNA NP. In vivo antigen-specific lysis of NP peptide-loaded target cells was evaluated in lymph nodes and spleens 4 or 5 days after the last immunization. **d** Duration of T-cell responses in mice immunized twice with 17 µg of NP mRNA with 1 week between vaccinations according to schedule 3. Antigen-specific lysis of NP peptide-loaded target cells was assessed by flow cytometry and IFN-γ production was assessed by ELISPOT after boost vaccination on the indicated days. Results are shown as median (**a**–**c**) or median ± IQR (**d**) with each symbol representing one mouse for a total of 4–10 mice per experiment. Differences between groups were analyzed by Kruskal–Wallis test with Bonferroni correction

To further investigate the efficiency of intranodal mRNA vaccination, we evaluated whether we could lower the administered dose and reduce the number of immunizations. To this end, mice were immunized according to several immunization schedules with varying doses of NP mRNA (Fig. 2a–c). A single immunization with 50 µg of NP mRNA induced cytotoxic T-cell responses in both lymph nodes and, to a lesser extent, the spleen (Fig. 2a), but analysis of these responses suggests that they were less potent compared to the results obtained from triple immunization with 50 µg NP mRNA in the previous experiment. In comparison, analysis of cytotoxic T-cell responses after vaccination with 50 µg of NP mRNA and 2 weeks between immunizations suggests that this prime-boost schedule improves targeted T-cell responses (Fig. 2b). Furthermore, the data also shows that T-cell responses in the spleen were higher than those in the lymph nodes, demonstrating that this schedule induces better systemic immunity.

Next, we assessed whether we could lower the administered dose of mRNA. To this end, mice were injected intranodally with 17 µg of NP mRNA according to a prime-boost schedule (schedule 3). Instead of a 2-week interval between immunizations, we shortened the time to 1 week as shorter time-intervals were shown to improve T-cell responses [28]. Analysis of cytotoxic T-cell responses in lymph nodes and spleens after this immunization schedule suggests that these were comparable to those achieved through prime-boost immunization with 50 µg NP mRNA (schedule 2), though the data suggests a higher variation between animals (Fig. 2c).

We further evaluated the potency of this vaccination schedule (schedule 3) by investigating the duration of active T-cell responses and found that cytotoxic T-cell responses capable of lysing antigen-pulsed target cells were still elevated 21 days after booster immunization in both lymph nodes and spleen (Fig. 2d). Although cytolytic responses were significantly reduced on day 28, ELISPOT analysis demonstrated the persistence of IFN-γ producing T cells in the spleen at this timepoint. On day 49, both cytolytic and IFN-γ T-cell responses subsided.

Together, these data demonstrate that intranodal prime-boost immunization with a low-dose mRNA vaccine induces robust and prolonged T-cell responses against NP.

### Intranodal immunization with NP mRNA results in viral clearance and modest protection against a heterologous challenge

**Fig. 3:**
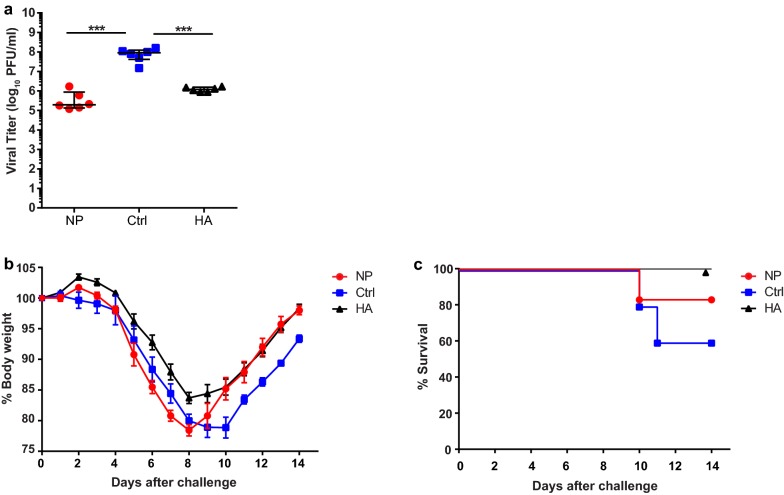
Protective efficacy of intranodal immunizations of NP mRNA against heterologous challenge. BALB/c mice were immunized twice with 17 µg of NP or control mRNA (Ctrl) or 50 µg of HA mRNA with 1 week between immunizations. 8 weeks after boost immunizations, mice were infected with 1xLD50 of influenza A H1N1 PR8. **a** Determination of viral titers in the BALF isolated on day 6 after infection. Results are shown as median ± IQR and each symbol represents one mouse (*n *= 6 per group). Differences were determined by one-way ANOVA. **b** Body weight changes after challenge expressed as mean ± SEM. Upon reaching the human-endpoint, mice were excluded from the data. **c** Survival of mice after challenge (*n *= 6 for NP and HA groups, *n *= 5 for control mice)

Next, we evaluated whether intranodally administered mRNA encoding NP could protect mice from a lethal challenge with a heterologous strain of influenza. Mice were immunized twice with 17 µg of NP mRNA or control mRNA and infected 8 weeks later with influenza A H1N1 PR8. As a positive control, we included mice immunized with mRNA encoding PR8 HA. Both NP and HA vaccinated animals showed significantly lower viral titers within the lungs on day 6 after challenge compared to control animals (Fig. 3a). However, both HA and NP vaccinated animals experienced temporary weight loss, which was more pronounced in the NP conditions (Fig. 3b). Compared to control animals, NP vaccinated mice showed a trend towards greater weight loss from day 4 to day 8, but their weight recovered faster after day 8. In total, 40% of control mice succumbed to the infection compared to 20% of NP vaccinated mice (Fig. 3c). These data show that intranodal immunization with NP mRNA contributes to viral clearance, but provides only modest protection against heterologous H1N1 infection.

### Analysis of immune cell infiltrates in spleen and BALF of vaccinated animals

As intranodal vaccination with NP encoding mRNA resulted in modest protection against influenza A virus challenge and a temporary increase in weight loss compared to control mice, we performed additional immune analyses to identify the underlying mechanisms.

**Fig. 4:**
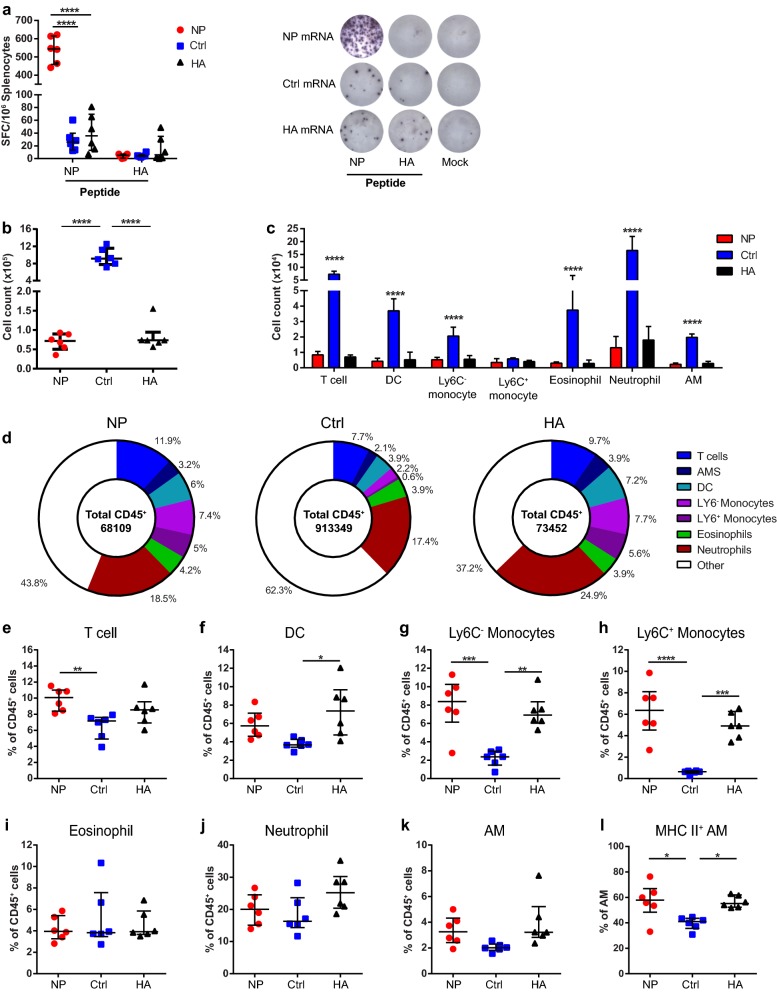
Analysis of systemic responses and immune cell populations in the BALF after H1N1 challenge. BALB/c mice were immunized twice with 17 µg of NP or control mRNA (Ctrl) or 50 µg of HA mRNA with 1 week between immunizations. 8 weeks after boost immunizations, mice (*n *= 6/group) were infected with 1xLD50 of influenza A H1N1 PR8. On day 6 after challenge, spleens and BALF were isolated. **a** IFN-γ ELISPOT on splenocytes isolated from vaccinated animals on day 6 after challenge and re-stimulated with the indicated peptides. Images of wells are representative for all mice for the respective conditions. **b**–**l** Flow cytometric analysis of immune cell populations within the BALF. AM, Alveolar macrophages. **b** Absolute cell count of CD45^+^ cells. **c** Absolute cell count of CD45^+^ subsets. **d** Donut-charts showing the composition of CD45^+^ subsets per group. Fractions show the median of the indicated subpopulations. The number in the center shows the median number of CD45^+^ cells for that group. **e**–**k** Percentages of CD45^+^ subsets within the CD45^+^ population. **l** Percentage of MHC II expressing AM within the general AM population. For all graphs, except donut-charts, results are shown as median ± IQR and each symbol represents one mouse for a total of 6 mice per group. Differences between groups were calculated using one-way ANOVA

First, we assessed whether infection by H1N1 results in re-activation of systemic antigen-specific T-cell responses in vaccinated animals. To this end, IFN-γ ELISPOT analysis was performed on the spleens of vaccinated animals on day 6 after infection. Consistent with the previous results, we found significantly higher responses against NP in NP mRNA vaccinated animals, compared to HA and control vaccinated animals, although responses against NP were also found in the latter groups (Fig. 4a). Furthermore, HA immunized animals did not induce robust IFN-γ T-cell responses against HA peptide. HA-peptide responses were also undetectable in NP immunized or control vaccinated animals. These results are consistent with NP being an immunodominant target for CD8^+^ T-cell responses and demonstrate that intranodal immunization with NP induces T-cell responses which are re-activated upon heterologous infection.

As we found a reduction in lung viral titers, we investigated the composition of the immune cell infiltrate in the BALF of vaccinated animals on day 6 after infection. Flow cytometry was used to distinguish T cells, DCs, monocytes, eosinophils, neutrophils and alveolar macrophages (AMs) in the BALF (see Additional file 1: Figure S1). While NP and HA mice had similar numbers of CD45^+^ cells in the BALF, the BALF of control animals contained a more than tenfold higher number of these cells (Fig. 4b). In line with this result, we found significantly higher numbers of T cells, DCs, Ly6C^−^ monocytes, eosinophils, neutrophils and AMs, but not Ly6C^+^ monocytes, in the BALF of control animals (Fig. 4c).

However, analysis of the relative composition of infiltrating CD45^+^ cells revealed a more nuanced image between groups (Fig. 4d). Mice vaccinated with NP mRNA showed an increased percentage of T cells within the CD45^+^ population compared to the control group (Fig. 4e). In the HA group, increased percentages of DCs were found compared to control mice (Fig. 4f). Both NP and HA vaccinated animals also showed significantly increased percentages of Ly6C^−^ and Ly6C^+^ monocytes compared to controls (Fig. 4g, h).

As AMs can contribute to T-cell responses through antigen presentation, we also assessed the expression of MHC II on this subset. Although no differences were found in the percentages of the overall AM populations in the BALF, for both NP and HA animals an increase in the percentage of AMs expressing MHC II was observed (Fig. 4l). No significant increases were found for the percentage of eosinophils and neutrophils between groups, though control group animals displayed increased populations within the granulocyte gate which were neither eosinophils or neutrophils (included in the ‘Other’ group in Fig. 4d, data not shown).

To summarize, these data show that NP vaccination induces systemic T-cell responses which can be re-activated and are present in the lungs during heterologous infection. In addition, NP vaccination results in lower numbers of infiltrating immune cells in the BALF and also affects the composition of innate cells.

### Analysis of cytokines in the BALF of vaccinated animals

To further explore the differences observed by flow cytometry, we evaluated the inflammatory cytokine profiles in the lungs of vaccinated animals.

**Fig. 5:**
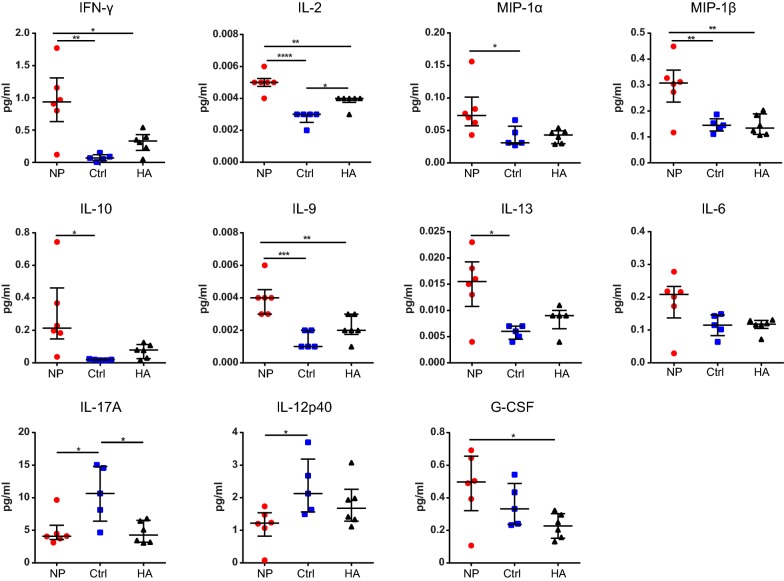
Analysis of cytokines in the BALF of vaccinated animals after H1N1 challenge. BALB/c mice were immunized twice with 17 µg of NP or control mRNA (Ctrl) or 50 µg of HA mRNA with 1 week between immunizations. 8 weeks after boost immunizations, mice were infected with 1xLD50 of influenza A H1N1 PR8. BALF was isolated on day 6 after challenge and cytokines were analyzed using Luminex. Graphs depict the concentration of the indicated cytokines as median ± IQR with each symbol representing one mouse. *n *= 6 for NP and HA groups, *n *= 5 for Ctrl group. Differences between groups were calculated using one-way ANOVA

Of the 23 cytokines and chemokines that were analyzed by Luminex assay (see Additional file 2: Table S1), significantly higher concentrations were observed for the proinflammatory factors IFN-γ, IL-2, MIP-1α/CCL3, MIP-1β/CCL4, IL-9, IL-13 and the anti-inflammatory cytokine IL-10 for NP vaccinated animals on day 6 after infection compared to control animals (Fig. 5). In addition, a strong trend towards higher concentrations of IL-6 was observed. Compared to HA recipients, NP vaccinated animals showed elevated levels of IFN-γ, IL-2, IL-9 and G-CSF. In contrast, increased concentrations of IL-17A and IL-12p40 were found in the BALF of control mice.

These results show that upon infection, intranodal vaccination with NP mRNA enhances the production of a distinct cytokine profile, which mostly bears a proinflammatory signature.

## Discussion

In this study, we show that intranodal injection of a low dose naked mRNA vaccine encoding influenza NP elicits robust T-cell responses and is capable of protecting mice against heterologous infection. Although initially a slightly enhanced weight loss was observed, NP mRNA vaccinated recipients recovered faster from infection.

Nucleic acid vaccines represent a promising strategy against pathogens owing to their versatility in encoding any protein or antigen of choice. In addition, such vaccines can be manufactured using the same materials and processes irrespective of the encoded protein, providing the potential for rapid and flexible vaccine production and economies of scale [29]. Despite these benefits, DNA vaccines have important safety concerns such as the long-term presence and expression of plasmid DNA, albeit at low levels [30]. Furthermore, low levels of plasmid integration into the host’s genome have been detected, harboring the risk of insertional mutagenesis [31, 32]. In contrast, mRNA does not enter the nucleus and therefore does not integrate into DNA by itself [33, 34]. As mRNA does not need to pass the nuclear membrane, its translation is almost immediate upon cytosol entry and is also efficient in non-dividing cells [35, 36]. Importantly, the expression of mRNA is also transient which limits the risk of toxicity [37].

Our group previously reported that intranodal immunization using naked mRNA induces strong primary T-cell responses and memory formation in mice [15, 38]. Consistent with these data, intranodal immunization with mRNA encoding NP derived from influenza A H3N2 induced strong and systemic T-cell responses which remained active for several weeks before subsiding, even at low doses of the vaccine. Furthermore, responses induced through this method proved to be superior compared to those induced by intramuscular DNA vaccination.

Several groups have demonstrated the induction of T-cell responses and protective efficacy after intramuscular vaccination with DNA NP in preclinical studies [27, 39–45]. We therefore wanted to use this well-established model for nucleotide vaccination to benchmark our intranodal mRNA vaccine approach. While the purpose of this study was not to extensively compare both vaccination methods, two factors could explain our results. First, the intranodal delivery route has been shown to be superior for both mRNA and DNA methods in eliciting antigen-specific T-cell responses [15, 16, 46]. In this regard, intranodal DNA immunization allowed for a 100-fold lower vaccine dose while maintaining similar CD8^+^ T-cell responses compared to intramuscular delivery [16]. Another potential factor is the immunogenicity of the molecules. Though DNA has shown potential in preclinical models, the translation of these results into humans has been challenging in terms of achieving sufficient immunogenicity [47]. In this regard, mRNA has been shown to possess intrinsic adjuvant activity which facilitates the induction of adaptive responses [46] via the triggering of different RNA sensors and type I IFN induction [48, 49]. Indeed, intratumoral delivery of ‘control’ mRNA encoding irrelevant antigens was shown to reduce tumor growth by itself [50].

It is important to note, however, that DNA also activates immunostimulatory pathways via pathogen recognition receptors including TLR9 [51], TBK1 [52], AIM2 [53] and the cGAS-STING pathway [54].

Upon intranodal vaccination, the effector T-cell response was sustained for several weeks before subsiding. Though, the duration and quality of a vaccine-induced immune response is dependent on many factors including the antigen, early studies in acute lymphocytic choriomeningitis virus (LCMV) infection revealed that the peak of the T-cell response occurs at day 8 post-infection followed by the contraction phase during which 90% of T cells die by day 21 [55]. In a tumor mouse model, T-cell responses induced by intranodal mRNA vaccination were almost fully contracted around day 24 [38]. In contrast, we still observed elevated IFN-γ producing T cell responses in the spleen at 28 days after the last immunization suggesting that the effector T-cell response had not yet fully contracted.

Upon heterologous H1N1 infection, these responses were systemically re-activated as we found increased IFN-γ ^+^ T-cell responses in the spleens of NP vaccinated animals. However, splenic CD8^+^ T cells were shown to be negligible for protection whereas T-cell responses in the lungs were crucial for live-attenuated influenza vaccine-induced protection [56]. It is therefore unclear to which extent these systemic responses contributed to protection which could explain the relatively modest protection against influenza A virus challenge infection.

Nevertheless, NP mRNA vaccination lowered lung viral titers which is consistent with earlier reports from our group that intranodal mRNA can establish mucosal T-cell responses in the lungs [38]. Consistent with the notion that T-cell based vaccines clear infected cells but do not completely prevent disease symptoms [57, 58], we found that NP vaccinated mice initially experienced a greater weight loss after infection compared to control animals.

Immunopathology from excessive inflammation has been widely described in the pathology of influenza, suggesting that protective immunity is a fine balance between offense and restraint. In this regard, CD8^+^ T-cell responses capable of killing infected cells can significantly enhance viral clearance and speed up recovery, but can also contribute to destructive lung inflammation when left unchecked [57, 59]. This phenomenon was demonstrated in a T-cell deficient mouse model in which infection with influenza resulted in slower disease progression and lung pathology compared to wildtype mice. However, progression lasted much longer due to sustained viral replication ultimately leading to significantly lower survival of T-cell deficient mice [60]. In line with these studies, we observed that mice vaccinated with NP mRNA initially experienced greater weight loss, but a faster recovery later on. Analysis of the BALF of NP vaccinated mice showed a larger representation of T cells and monocytes within the CD45^+^ population compared to control animals. Strikingly, however, control mice displayed significantly higher absolute numbers of leukocytes within the BALF which has previously correlated with unchecked inflammation and disease progression in influenza [61, 62]. Although NP vaccinated mice had lower total T-cell numbers in the BALF compared with challenged control mRNA vaccinated mice, these T cells seemed to be more potent in viral clearance.

Ly6C^−^ monocytes are considered anti-inflammatory and play a role in tissue repair, but have been shown to contribute to early inflammation and attraction of neutrophils [63]. In contrast, Ly6C^+^ monocytes induce a more pronounced inflammatory response through cytokine secretion and contribute to pathogen clearance via phagocytosis. Interestingly of all cell types analyzed, only Ly6C^+^ monocytes were present in equal numbers in both NP, HA and control groups. Considering that monocytes are a major target for influenza [64] and undergo apoptosis after infection [65], it is possible that the lack of viral control resulted in increased cell death and thus lower numbers within the BALF of challenged control mRNA vaccinated mice. Although we found no studies investigating the preference of influenza to target Ly6C^+^ over Ly6C^−^ monocytes, it was shown that IL-17 modulates the transition of Ly6C^+^ to Ly6C^−^ monocytes [66]. This could explain why Ly6C^−^ monocytes were overrepresented in control mice compared to Ly6C^+^ subsets.

Despite lower leukocyte recruitment, NP vaccinated mice showed elevated concentrations of proinflammatory cytokines within the lungs, indicating enhanced immune cell activation and inflammation compared to control animals. Though certain cytokines have been correlated with protection [67], their exact roles remain to be fully elucidated [68]. Furthermore, high levels of inflammatory cytokines have previously been directly correlated with symptom scores in infected patients and could therefore explain the initial weight loss [69, 70]. Hence, more research will be needed to understand the context-specific roles of cytokines during influenza infection.

An important caveat of interpreting these results is the lack of mRNA studies utilizing NP as a vaccine antigen against influenza. Petsch et al. demonstrated that intradermal administration of protamine-complexed mRNA encoding NP induced T-cell-dependent heterologous protection against influenza with a lower loss of weight compared to our study [71]. While no data was provided on cytokines or infiltrating immune cell populations in this study, we can hypothesize that this vaccination strategy was associated with a lower T-cell induced immunopathology. This is based on previous research by our group which showed that intradermal immunization with mRNA leads to lower T-cell responses compared to the intranodal route [15].

Compared to this study, we used shorter time intervals between immunizations as it has been suggested that short frequency immunizations can improve vaccine responses [72, 73]. In this regard, the speed of achieving adequate immunity could be crucial during outbreaks when time is of the essence. We were also able to use lower doses of mRNA which was likely facilitated by using the intranodal route [16].

Though less practical than intramuscular vaccines, intranodal vaccination provides the potential for lower vaccine doses while maintaining immunogenicity and allows for ‘naked’ mRNA delivery without packaging. In a clinical setting, intranodal mRNA delivery can be carried out via ultrasound-guided syringe injections in the inguinal lymph nodes, which was demonstrated to be safe and immunogenic in a phase I mRNA vaccine study for HIV [74]. Importantly, intranodal delivery of personalized mRNA vaccines significantly reduced metastatic events and was associated with sustained progression-free survival in melanoma patients [75]. In the context of an universal vaccine against influenza, the more labor-intensive process of intranodal vaccination could be justified as an ideal universal vaccine provides protection against multiple influenza strains for multiple seasons. In this regard, our results show that, in mice, potent T-cell responses can be induced, which remain active for several weeks before subsiding and are reactivated upon infection, indicating T-cell memory. Compared to the study by Petsch et al, the time between the last immunization and viral challenge was longer in our setting due to the length of active T-cell responses. However, further research must be conducted to improve protection and prevent the initial loss of weight by vaccination.

## Conclusion

In conclusion, intranodal immunization with mRNA encoding NP induces strong T-cell responses capable of heterologous viral clearance within the lungs and improving recovery from infection. However, this response does result in a more pronounced, but transient, weight loss which occurs early on during infection most likely due to exaggerated inflammation. In this regard, T cells are a powerful target for broadly protective responses but must be balanced in order to avoid enhanced disease progression through pulmonary injury. The data provided in this report help to support the resolution of this paradox and pave the way towards a universal influenza vaccine.

## Additional files

**Additional file 1: Figure S1.** Gating strategy for the flow cytometric analysis of CD45^+^ subsets within the BALF. Debris and doublets were first excluded based on forward scatter (FSC) and sideward scatter (SSC). Dead cells were then excluded based on positivity for the viability dye following the identification of CD45^+^ cells through staining. A sequential gating strategy was then used to identify T cells, B cells, alveolar macrophages (AM), dendritic cells (DC), neutrophils, eosinophils and Ly6C^+^ and Ly6C^−^ monocytes according to the markers described in Additional file 2: Table S1. T cells and B cells were first isolated based on positivity for CD3 or CD19 and negativity for CD11c. As these antibodies were conjugated with the same fluorochrome, T cells and B cells were further distinguished based on negative for MHC II or positive for MHC II, respectively. AM were then isolated based on high expression of Siglec F and CD11c. MHC II^+^ AM were then isolated within the AM population based on MHC II expression. Next, DCs were isolated based on expression of CD11c and MHC II. MHC II negative cells were further distinguished using SSC-A into granulocyte (SSC-A high) or monocyte (SSC-A low) subsets. Within the granulocyte gate, eosinophils were separated based on expression of Siglec F and high expression of CD11b. Neutrophils were distinguished based on high expression of CD11b, Siglec F negativity and Ly6G positivity. For monocytes, absence of Siglec F and Ly6G was first confirmed, following the separation of monocyte subsets based on the presence of Ly6C.

**Additional file 2: Table S1.** Concentrations of cytokines within the BALF of vaccinated animals infected with H1N1 PR8 influenza. Values show the median concentration (pg/ml) with interquartile range (Q1–Q3) of the indicated cytokine or chemokine for the respective group. ND, not detected.

## Data Availability

The datasets used and/or analysed during the current study are available from the corresponding author on reasonable request.

## Abbreviations

NP: nucleoprotein
mRNA: messenger RNA
MHC: major histocompatibility complex
NA: neuraminidase
TLR: toll-like receptor
RLR: RIG-I-like receptors
DC: dendritic cell
LNP: lipid nanoparticle
tNGFR: truncated nerve growth factor
BALF: bronchoalveolar lavage fluid
IFN-γ: interferon γ
SFC: spot-forming cells
CTV: cell-trace violet
AM: alveolar macrophage
IL: interleukin
MIP-1α: macrophage inflammatory protein 1α
MIP-1β: macrophage inflammatory protein 1β
G-CSF: granulocyte colony-stimulating factor
M1: matrix protein 1
M2: matrix protein 2
M2e: matrix protein 2 ectodomain
LCMV: lymphocytic choriomeningitis virus

## References

[1] Demicheli V, Jefferson T, Al-Ansary LA, Ferroni E, Rivetti A, Di Pietrantonj C (2014). Vaccines for preventing influenza in healthy adults. Cochrane
Database Syst Rev.

[2] Carrat F, Flahault A (2007). Influenza vaccine: the challenge of antigenic drift. Vaccine.

[3] Uhart M, Bricout H, Clay E, Largeron N (2016). Public health and economic impact of seasonal influenza vaccination with quadrivalent influenza vaccines compared to trivalent influenza vaccines in Europe. Hum Vaccine Immunother.

[4] Zambon MC (1999). Epidemiology and pathogenesis of influenza. J Antimicrob Chemother.

[5] Gerdil C (2003). The annual production cycle for influenza vaccine. Vaccine.

[6] Krammer F, García-Sastre A, Palese P (2018). Is it possible to develop a “universal” influenza virus vaccine?. Cold Spring Harb Perspect Biol..

[7] McMichael AJ, Gotch FM, Noble GR, Beare PAS (2010). Cytotoxic T-cell immunity to influenza. N Engl J Med.

[8] Topham DJ, Tripp RA, Doherty PC (1997). CD8+ T cells clear influenza virus by perforin or Fas-dependent processes. J Immunol..

[9] Boon ACM, de Mutsert G, van Baarle D, Smith DJ, Lapedes AS, Fouchier RAM (2004). Recognition of homo- and heterosubtypic variants of influenza A viruses by human CD8+ T lymphocytes. J Immunol..

[10] Kreijtz JHCM, de Mutsert G, van Baalen CA, Fouchier RAM, Osterhaus ADME, Rimmelzwaan GF (2008). Cross-recognition of avian H5N1 influenza virus by human cytotoxic T-lymphocyte populations directed to human influenza A virus. J Virol.

[11] Gras S, Kedzierski L, Valkenburg SA, Laurie K, Liu YC, Denholm JT (2010). Cross-reactive CD8+ T-cell immunity between the pandemic H1N1-2009 and H1N1-1918 influenza A viruses. Proc Natl Acad Sci USA.

[12] Hillaire MLB, Vogelzang-van Trierum SE, Kreijtz JHCM, de Mutsert G, Fouchier RAM, Osterhaus ADME (2013). Human T-cells directed to seasonal influenza A virus cross-react with 2009 pandemic influenza A (H1N1) and swine-origin triple-reassortant H3N2 influenza viruses. J Gen Virol..

[13] Pardi N, Hogan MJ, Porter FW, Weissman D (2018). mRNA vaccines—a new era in vaccinology. Nat Rev Drug Discov..

[14] Edwards DK, Jasny E, Yoon H, Horscroft N, Schanen B, Geter T (2017). Adjuvant effects of a sequence-engineered mRNA vaccine: translational profiling demonstrates similar human and murine innate response. J Transl Med..

[15] Van Lint S, Goyvaerts C, Maenhout S, Goethals L, Disy A, Benteyn D (2012). Preclinical evaluation of TriMix and antigen mRNA-based antitumor therapy. Cancer Res.

[16] Maloy KJ, Erdmann I, Basch V, Sierro S, Kramps TA, Zinkernagel RM (2001). Intralymphatic immunization enhances DNA vaccination. Proc Natl Acad Sci USA.

[17] Senti G, Kündig TM (2015). Intralymphatic immunotherapy. World Allergy Organ J..

[18] Johansen P, Häffner AC, Koch F, Zepter K, Erdmann I, Maloy K (2005). Direct intralymphatic injection of peptide vaccines enhances immunogenicity. Eur J Immunol.

[19] Voeten JT, Bestebroer TM, Nieuwkoop NJ, Fouchier RA, Osterhaus AD, Rimmelzwaan GF (2000). Antigenic drift in the influenza A virus (H3N2) nucleoprotein and escape from recognition by cytotoxic T lymphocytes. J Virol.

[20] Van Nuffel AMT, Benteyn D, Wilgenhof S, Pierret L, Corthals J, Heirman C (2012). Dendritic cells loaded with mRNA encoding full-length tumor antigens prime CD4+ and CD8+ T cells in melanoma patients. Mol Ther.

[21] Bonehill A, Heirman C, Tuyaerts S, Michiels A, Breckpot K, Brasseur F (2004). Messenger RNA-electroporated dendritic cells presenting MAGEA3 simultaneously in HLA class I and class II molecules. J Immunol..

[22] Van der Jeught K, De Koker S, Bialkowski L, Heirman C, Tjok Joe P, Perche F (2018). Dendritic cell targeting mRNA lipopolyplexes combine strong antitumor T-cell immunity with improved inflammatory safety. ACS Nano..

[23] Verhelst J, Parthoens E, Schepens B, Fiers W, Saelens X (2012). Interferon-inducible protein Mx1 inhibits influenza virus by interfering with functional viral ribonucleoprotein complex assembly. J Virol.

[24] Van Hoecke L, Job ER, Saelens X, Roose K (2017). Bronchoalveolar lavage of murine lungs to analyze inflammatory cell infiltration. J Vis Exp..

[25] Lambe T, Carey JB, Li Y, Spencer AJ, Van Laarhoven A, Mullarkey CE (2013). Immunity against heterosubtypic influenza virus induced by adenovirus and MVA expressing nucleoprotein and matrix protein-1. Sci Rep.

[26] Guardo AC, Joe PT, Miralles L, Bargalló ME, Mothe B, Krasniqi A (2016). Preclinical evaluation of an mRNA HIV vaccine combining rationally selected antigenic sequences and adjuvant signals (HTI-TriMix). AIDS..

[27] Lee LYY, Izzard L, Hurt AC (2018). A review of DNA vaccines against influenza. Front Immunol..

[28] Bins AD, Jorritsma A, Wolkers MC, Hung C-F, Wu T-C, Schumacher TNM (2005). A rapid and potent DNA vaccination strategy defined by in vivo monitoring of antigen expression. Nat Med.

[29] Rauch S, Jasny E, Schmidt KE, Petsch B (2018). New vaccine technologies to combat outbreak situations. Front Immunol Front.

[30] Armengol G, Ruiz LM, Orduz S (2004). The injection of plasmid DNA in mouse muscle results in lifelong persistence of DNA, gene expression, and humoral response. Appl Biochem Biotechnol Part B Mol Biotechnol..

[31] Wang Z, Troilo PJ, Wang X, Griffiths TG, Pacchione SJ, Barnum AB (2004). Detection of integration of plasmid DNA into host genomic DNA following intramuscular injection and electroporation. Gene Ther.

[32] Schalk JA, Mooi FR, Berbers GA, Aerts LA, Ovelgönne H, Kimman TG (2006). Preclinical and clinical safety studies on DNA vaccines. Hum Vaccines..

[33] Sahin U, Karikó K, Türeci Ö (2014). mRNA-based therapeutics—developing a new class of drugs. Nat Rev Drug Discov..

[34] Maruggi G, Zhang C, Li J, Ulmer JB, Yu D (2019). mRNA as a transformative technology for vaccine development to control infectious diseases. Mol Ther..

[35] Wolff JA, Malone RW, Williams P, Chong W, Acsadi G, Jani A (1990). Direct gene transfer into mouse muscle in vivo. Science.

[36] Zou S, Scarfo K, Nantz MHH, Hecker JGG (2010). Lipid-mediated delivery of RNA is more efficient than delivery of DNA in non-dividing cells. Int J Pharm.

[37] Vallazza B, Petri S, Poleganov MA, Eberle F, Kuhn AN, Sahin U (2015). Recombinant messenger RNA technology and its application in cancer immunotherapy, transcript replacement therapies, pluripotent stem cell induction, and beyond. Wiley Interdiscip Rev RNA.

[38] Bialkowski L, van Weijnen A, Van der Jeught K, Renmans D, Daszkiewicz L, Heirman C (2016). Intralymphatic mRNA vaccine induces CD8 T-cell responses that inhibit the growth of mucosally located tumours. Sci Rep.

[39] Khan KH (2013). DNA vaccines: roles against diseases. Germs..

[40] Epstein SL, Tumpey TM, Misplon JA, Lo CY, Cooper LA, Subbarao K (2002). DNA vaccine expressing conserved influenza virus proteins protective against H5N1 challenge infection in mice. Emerg Infect Dis..

[41] Jimenez GS, Planchon R, Wei Q, Rusalov D, Geall A, Enas J (2007). Vaxfectin™-formulated influenza DNA vaccines encoding NP and M2 viral proteins protect mice against lethal viral challenge. Hum Vaccine.

[42] Ulmer JB, Donnelly JJ, Parker SE, Rhodes GH, Felgner PL, Dwarki VJ (1993). Heterologous protection against influenza by injection of DNA encoding a viral protein. Science.

[43] Wang W, Li R, Deng Y, Lu N, Chen H, Meng X (2015). Protective efficacy of the conserved NP, PB1, and M1 proteins as immunogens in DNA- and vaccinia virus-based universal influenza A virus vaccines in mice. Clin Vaccine Immunol.

[44] Chang H, Huang C, Wu J, Fang F, Zhang W, Wang F (2010). A single dose of DNA vaccine based on conserved H5N1 subtype proteins provides protection against lethal H5N1 challenge in mice pre-exposed to H1N1 influenza virus. Virol J.

[45] Ulmer JB, Fu TM, Deck RR, Friedman A (1998). Protective CD4+ and CD8+ T cells against influenza virus induced by vaccination with nucleoprotein DNA. J Virol.

[46] Kreiter S, Selmi A, Diken M, Koslowski M, Britten CM, Huber C (2010). Intranodal vaccination with naked antigen-encoding RNA elicits potent prophylactic and therapeutic antitumoral immunity. Cancer Res.

[47] Li L, Petrovsky N (2016). Molecular mechanisms for enhanced DNA vaccine immunogenicity. Expert Rev Vaccines.

[48] Karikó K, Muramatsu H, Ludwig J, Weissman D (2011). Generating the optimal mRNA for therapy: HPLC purification eliminates immune activation and improves translation of nucleoside-modified, protein-encoding mRNA. Nucleic Acids Res.

[49] Weissman D, Karikó K (2015). mRNA: fulfilling the promise of gene therapy. Mol Ther.

[50] Van der Jeught K, Joe PT, Bialkowski L, Heirman C, Daszkiewicz L, Liechtenstein T (2014). Intratumoral administration of mRNA encoding a fusokine consisting of IFN-β and the ectodomain of the TGF-β receptor II potentiates antitumor immunity. Oncotarget..

[51] Rottembourg D, Filippi CM, Bresson D, Ehrhardt K, Estes EA, Oldham JE (2010). Essential role for TLR9 in prime but not prime-boost plasmid DNA vaccination to activate dendritic cells and protect from lethal viral infection. J Immunol.

[52] Ishii KJ, Kawagoe T, Koyama S, Matsui K, Kumar H, Kawai T (2008). TANK-binding kinase-1 delineates innate and adaptive immune responses to DNA vaccines. Nature.

[53] Schroder K, Muruve DA, Tschopp J (2009). Innate immunity: cytoplasmic DNA sensing by the AIM2 inflammasome. Curr Biol.

[54] Sun L, Wu J, Du F, Chen X, Chen ZJ (2013). Cyclic GMP-AMP synthase is a cytosolic DNA sensor that activates the type I interferon pathway. Science.

[55] Murali-Krishna K, Altman JD, Suresh M, Sourdive DJD, Zajac AJ, Miller JD (1998). Counting antigen-specific CD8 T cells: a reevaluation of bystander activation during viral infection. Immunity..

[56] Lau Y-F, Wright AR, Subbarao K (2012). The contribution of systemic and pulmonary immune effectors to vaccine-induced protection from H5N1 influenza virus infection. J Virol..

[57] Duan S, Thomas PG (2016). Balancing immune protection and immune pathology by CD8+ T-cell responses to influenza infection. Front Immunol..

[58] Pendzialek J, Roose K, Smet A, Schepens B, Kufer P, Raum T (2017). Bispecific T cell engaging antibody constructs targeting a universally conserved part of the viral M2 ectodomain cure and prevent influenza A virus infection. Antiviral Res.

[59] Tavares LP, Teixeira MM, Garcia CC (2017). The inflammatory response triggered by influenza virus: a two edged sword. Inflamm Res.

[60] Wells MA, Albrecht P, Ennis FA (1981). Recovery from a viral respiratory infection. I. Influenza pneumonia in normal and T-deficient mice. J Immunol..

[61] Perrone LA, Plowden JK, García-Sastre A, Katz JM, Tumpey TM (2008). H5N1 and 1918 pandemic influenza virus infection results in early and excessive infiltration of macrophages and neutrophils in the lungs of mice. PLoS Pathog..

[62] La Gruta NL, Kedzierska K, Stambas J, Doherty PC (2007). A question of self-preservation: immunopathology in influenza virus infection. Immunol Cell Biol.

[63] Auffray C, Fogg D, Garfa M, Elain G, Join-Lambert O, Kayal S (2007). Monitoring of blood vessels and tissues by a population of monocytes with patrolling behavior. Science.

[64] Hofmann P, Sprenger H, Kaufmann A, Bender A, Hasse C, Nain M (1997). Susceptibility of mononuclear phagocytes to influenza A virus infection and possible role in the antiviral response. J Leukoc Biol..

[65] Fesq H, Bacher M, Nain M, Gemsa D (1994). Programmed cell death (apoptosis) in human monocytes infected by influenza A virus. Immunobiology.

[66] Ge S, Hertel B, Susnik N, Rong S, Dittrich AM, Schmitt R (2014). Interleukin 17 receptor a modulates monocyte subsets and macrophage generation in vivo. PLoS ONE.

[67] Sridhar S, Begom S, Bermingham A, Hoschler K, Adamson W, Carman W (2013). Cellular immune correlates of protection against symptomatic pandemic influenza. Nat Med.

[68] Betakova T, Kostrabova A, Lachova V, Turianova L (2017). Cytokines induced during influenza virus infection. Curr Pharm Des..

[69] Hayden FG, Fritz R, Lobo MC, Alvord W, Strober W, Straus SE (1998). Local and systemic cytokine responses during experimental human influenza. A virus infection. Relation to symptom formation and host defense. J Clin Investig.

[70] Kaiser L, Fritz RS, Straus SE, Gubareva L, Hayden FG (2001). Symptom pathogenesis during acute influenza: interleukin-6 and other cytokine responses. J Med Virol.

[71] Petsch B, Schnee M, Vogel AB, Lange E, Hoffmann B, Voss D (2012). Protective efficacy of in vitro synthesized, specific mRNA vaccines against influenza A virus infection. Nat Biotechnol.

[72] Smith KA, Tam VL, Wong RM, Pagarigan RR, Meisenburg BL, Joea DK (2009). Enhancing DNA vaccination by sequential injection of lymph nodes with plasmid vectors and peptides. Vaccine..

[73] Heinzerling L, Basch V, Maloy K, Johansen P, Senti G, Wüthrich B (2006). Critical role for DNA vaccination frequency in induction of antigen-specific cytotoxic responses. Vaccine..

[74] Leal L, Guardo AC, Morón-López S, Salgado M, Mothe B, Heirman C (2018). Phase I clinical trial of an intranodally administered mRNA-based therapeutic vaccine against HIV-1 infection. AIDS.

[75] Sahin U, Derhovanessian E, Miller M, Kloke BP, Simon P, Löwer M (2017). Personalized RNA mutanome vaccines mobilize poly-specific therapeutic immunity against cancer. Nature..

