# Supplementary figures and images for: Intranodal administration of mRNA encoding nucleoprotein provides cross-strain immunity against influenza in mice
Source: J Transl Med. 2019 Jul 25;17:242. doi: 10.1186/s12967-019-1991-3 (PMC6659201; doi:10.1186/s12967-019-1991-3)

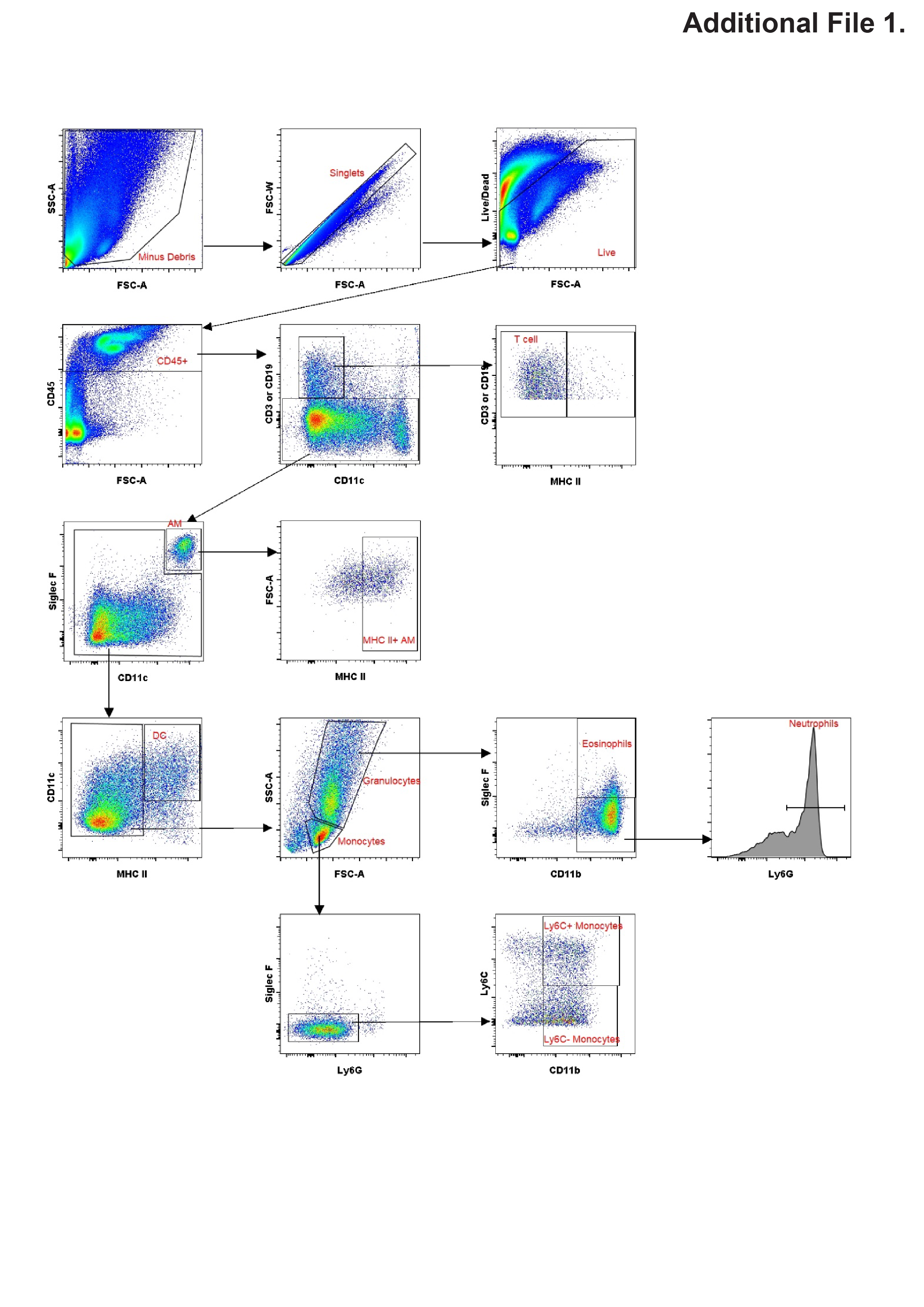

Supplement: Supplementary file 1 — Additional file 1: Figure S1. Gating strategy for the flow cytometric analysis of CD45+ subsets within the BALF. Debris and doublets were first excluded based on forward scatter (FSC) and sideward scatter (SSC). Dead cells were then excluded based on positivity for the viability dye following the identification of CD45+ cells through staining. A sequential gating strategy was then used to identify T cells, B cells, alveolar macrophages (AM), dendritic cells (DC), neutrophils, eosinophils and Ly6C+ and Ly6C− monocytes according to the markers described in Additional file 2: Table S1. T cells and B cells were first isolated based on positivity for CD3 or CD19 and negativity for CD11c. As these antibodies were conjugated with the same fluorochrome, T cells and B cells were further distinguished based on negative for MHC II or positive for MHC II, respectively. AM were then isolated based on high expression of Siglec F and CD11c. MHC II+ AM were then isolated within the AM population based on MHC II expression. Next, DCs were isolated based on expression of CD11c and MHC II. MHC II negative cells were further distinguished using SSC-A into granulocyte (SSC-A high) or monocyte (SSC-A low) subsets. Within the granulocyte gate, eosinophils were separated based on expression of Siglec F and high expression of CD11b. Neutrophils were distinguished based on high expression of CD11b, Siglec F negativity and Ly6G positivity. For monocytes, absence of Siglec F and Ly6G was first confirmed, following the separation of monocyte subsets based on the presence of Ly6C. [file 12967_2019_1991_MOESM1_ESM.tif]
